# Association between Chronic Pain and Sarcopenia in Greek Community-Dwelling Older Adults: A Cross-Sectional Study

**DOI:** 10.3390/healthcare12131303

**Published:** 2024-06-29

**Authors:** Maria Tsekoura, Evdokia Billis, Charalampos Matzaroglou, Elias Tsepis, John Gliatis

**Affiliations:** 1 Laboratory of Clinical Physiotherapy and Research, Department of Physiotherapy, School of Health Rehabilitation Sciences, University of Patras, 26504 Rio, Greece; 2 Department of Orthopaedics, School of Medicine, University of Patras, 26504 Patra, Greece

**Keywords:** chronic pain, sarcopenia, older adults

## Abstract

(1) Background: Sarcopenia and chronic pain are prevalent syndromes among older adults that negatively affect their quality of life. The present study aimed to investigate the relationship between chronic pain and sarcopenia among Greek community-dwelling older adults. (2) Methods: Older adults >60 years of age were enrolled in this descriptive, cross-sectional study. Sarcopenia status was assessed according to the EWGSOP2 2019 algorithm. This assessment included the evaluation of muscle strength, body composition and gait speed. Pain location and pain characteristics were assessed using a self-reported questionnaire. Pain severity was assessed via the visual analog scale. The participants were also asked to fill out the SARC-F, the Hospital Anxiety and Depression Scale (HADS) and the Falls Efficacy Scale-International (FES-I) questionnaire. (3) Results: This study included 314 participants with a mean age of 71.3 ± 7.4 years. The prevalence of sarcopenia was 19.4 (*n* = 61), and 44.26% of the sarcopenic participants recorded chronic pain. Chronic pain was associated with sarcopenia, comorbidities, the number of drugs and HADS. (4) Conclusions: The results demonstrated a high percentage of chronic pain in the sarcopenic population. The results also highlight the importance of the detection of chronic pain in older patients with sarcopenia in order to develop effective preventive and therapeutic strategies.

## 1. Introduction

Chronic pain in older adults is a worldwide problem [1]. Chronic pain is defined as pain that persists past the normal time of healing, usually characterized as pain for at least 3 months [2,3,4]. It is associated with disability, social isolation, premature death, cognitive decline and healthcare costs [4]. It is also associated with complications such as fall injuries and frailty [5], anxiety [6] and insomnia [7,8]. The prevalence of pain experience among the general population aged 65 years and older is over 50% [1,8,9]. With aging there is a rise in the incidence of chronic health conditions, leading to an increased burden of pain. According to the World Health Organization, musculoskeletal disorders are one of the leading causes of disability among older people [10]. The most common painful conditions affecting older adults are arthritis-related ones (e.g., chronic pain in the hip and knee or lower back pain) or cancer-related pain [5,11].

Sarcopenia is a progressive and generalized skeletal muscle disorder involving an accelerated decline in the quantity and quality of the skeletal muscle [12]. The prevalence of sarcopenia is high among older adults and has gradually been found to be associated with a range of negative health outcomes, such as functional decline, falls, physical limitations and disability, depression, institutionalization, a decrease in quality of life and mortality [13,14].

Scientific data show that sarcopenia has recently been associated with chronic pain. The results of a recent systematic review and meta-analysis provide evidence that the prevalence of sarcopenia is significantly higher in older adults with chronic pain as compared to those without pain [15]. However, the pathogenesis underlying the relationship between chronic pain and sarcopenia in older adults remains unknown. It has been hypothesized that pain potentially increases the risk for sarcopenia via factors such as immobility, sedentary behavior, depression, sleep problems, social isolation and malnutrition [16], as well as reduced activity and chronic inflammation.

The age of the global population is increasing rapidly and the size of the sarcopenic population is growing. The prevalence of sarcopenia in Greece has been reported at 13.2% among people ≥60 years of age, while the prevalence of probable sarcopenia is 25.4% [17]. However, the association between pain and sarcopenia has not been investigated. Pain among older adults is often under-recognized, under-reported and under-treated. Considering the high incidence of sarcopenia amongst older adults, it seems important to investigate the possibility of pain being a risk factor for sarcopenia and to, therefore, assess it effectively.

Given this background, the objective of the present study was to investigate the association between chronic pain and sarcopenia among community-dwelling, older adults. The evaluation of pain among patients with sarcopenia may provide important information relevant to sarcopenia prevention and health promotion and management.

## 2. Materials and Methods

This was a cross-sectional, analytical study that involved a total of 314 older adults living in Western Greece. The Ethics Committee of the University of Patras, Greece, approved the study protocol.

### 2.1. Participants

Participants were recruited from the University Hospital of Rio (outpatients), University of Patras, a private orthopedic clinic, and the Centers for the Elderly of the Achaia region in Greece.

Eligible participants had to be >60 years old and be able to read and understand the purpose of the study. Participants who had cognitive decline, identified through the Mini-Mental State Exam (with an MMSE score lower than 25 out of 30), were excluded from the study. The MMSE is a brief, reliable and valid quantitative measure of cognitive status in adults [18], extensively used in both clinical practice and research by various health professionals [19].

Exclusion criteria also included having a pacemaker fitted, patients with an amputated limb or a body mass index (BMI) > 50, the latter because of the requirements of the device measuring muscle mass (bioelectrical impedance analysis) [20].

### 2.2. Definition of Sarcopenia

Participants’ sarcopenia status was assessed according to the EWGSOP2 2019 algorithm, comprising three components: muscle strength, muscle mass and physical performance. Sarcopenia is probable when low muscle strength is detected (a reduced hand grip strength with values <16 kg and <27 kg was considered for females and males, respectively). A sarcopenia diagnosis is confirmed by the presence of low muscle mass (<5.5 kg/m^2^ in women; <7.0 kg/m^2^ in men). When low muscle strength, low muscle mass and low physical performance are all detected, sarcopenia is considered severe [12].

### 2.3. Outcome Measures

Assessment of older adults included evaluations of muscle strength, body composition and motor functions. Three validated questionnaires were also used for sarcopenia risk screening (SARC-F) [12] and to assess fear of falling (Falls Efficacy Scale-International) and anxiety and depression (Hospital Anxiety and Depression Scale).

### 2.4. Interview Survey

A self-reported questionnaire was used to obtain information from the participants about their socio-demographic characteristics (e.g., age, sex, marital status, number of children, past occupation, lifestyle habits, number of drugs used in the past year, comorbidities and history of falls in the past year).

### 2.5. Measurements

#### 2.5.1. Pain Assessment

Pain location and pain characteristics were assessed using a self-reported questionnaire. Pain was assessed according to the following questions: 1. In the last 12 months, have you experienced any body pains (no or yes) persisting for at least 3 months? 2. In which part(s) of your body do you feel this pain (head, neck, chest, stomach, shoulder, back, waist, buttocks, arm, leg, knees, wrist, fingers, ankle, toes)? Patients who reported pain completed the visual analog scale (VAS). The VAS is a pain rating scale of pain intensity and is a simple and reliable method usually used in clinical and research settings [21,22]. According to their pain status, participants were divided into the categories of no pain, single-site pain and more than one site of pain (multi-site pain).

#### 2.5.2. SARC-F

The EWGSOP2 algorithm incorporates the SARC-F questionnaire as a screening tool for sarcopenia [12]. SARC-F is a quick, inexpensive and convenient tool for sarcopenia self-reported risk screening [12,23]. It includes five questions (related to lifting and carrying a 10-pound weight, walking across a room, transferring from bed to chair, climbing a flight of 10 stairs, and frequency of falls in the past year) and is successfully adapted into Greek [23].

#### 2.5.3. Muscle Strength Assessment

Muscle strength was evaluated via hand grip strength (HGS). HGS was assessed with a calibrated hydraulic hand dynamometer (SAEHAN DHD-1, Seoul, Republic of Korea). Each participant was in a seated position with their knees and hips at 90°, with their elbow in flexion and their wrist in a neutral position. He/she squeezed the dynamometer with all of his/her strength using the dominant hand. Three measurements were performed, and the highest value was used for analysis [21,24].

#### 2.5.4. Body Composition Assessment

Muscle mass was assessed using bioelectrical impedance analysis (BIA) via a Tanita BC-601 model body analysis monitor. Participants stood on the scale barefoot (on two metallic electrodes) and held two metallic grip electrodes [20]. Fat-free mass (FFM) was measured using BIA, and skeletal muscle mass (SMM) was calculated via the following equation: SMM (kg) = 0.566 × FFM. Skeletal muscle mass index (SMMI) was calculated as skeletal muscle mass (kg)/height squared [25,26].

#### 2.5.5. Physical Performance

Walking speed was assessed via the four-meter (4 m) walking test. The participants walked this distance at a comfortable/habitual pace. A measure of gait speed over a 4 m distance was recorded. For the 4 m test, time in seconds was the recorded variable. The values lower than or equal to 0.8 m/s indicated low gait speed [12].

#### 2.5.6. Anxiety and Stress

The Greek version of the Hospital Anxiety and Depression Scale (HADS) was used to assess anxiety and depression among older adults [27]. It is a self-reported rating scale of 14 items (7 items for anxiety and 7 items for depression).

#### 2.5.7. Fear of Falling

The Falls Efficacy Scale-International (FES-I) was used to assess the level of concern about falling [28]. FES-1 is a 16-item, self-reported questionnaire, and has been translated and cross-culturally adapted into Greek. The Greek FES-I was found to be valid and reliable for use in rehabilitation research and clinical practice [29].

### 2.6. Data Analysis

SPSS 28.0 (SPSS, Chicago, IL, USA) was used for the statistical analysis. The descriptive characteristics were presented as the means ± standard deviations for quantitative variables and as the means of frequency for qualitative variables. The *T*-test for independent samples was used to determine the differences between participants with sarcopenia and participants without sarcopenia. Pearson’s r correlation coefficient was used to calculate the correlations between pain and other variables. Logistic regression analysis was used to identify the factors associated with chronic pain and sarcopenia. Statistical significance was accepted at *p* ≤ 0.05, and an adjusted odds ratio and 95% confidence interval were reported in order to consider the strength of association between the variables.

## 3. Results

The sample consisted of 314 older Greek adults aged between 60 and 95 years, with or without sarcopenia, and with a mean age of 71.3 ± 7.4 years. Participants‘ characteristics are presented in Table 1.

In face-to-face interviews with community-dwelling, older adults, 21% of the study participants reported having pain for at least three months. The results of our pain assessment of community-dwelling, older adults are presented in Table 2.

The results of the logistic regression analysis are presented in Table 3. Sarcopenia, comorbidities, the number of drugs and HADS were identified as strong predictors of chronic pain. No significant association between chronic pain and the other variables was recorded.

## 4. Discussion

To our knowledge, this is the first study to examine the association between chronic pain and sarcopenia among older Greek adults. Pain is a condition highly prevalent in older people [30,31]. The results of this study demonstrate (i) a high percentage of pain in the sarcopenic population and (ii) that sarcopenia, multimorbidity, the number of drugs and HADS were statistically significant variables in the logistic regression model. These results indicate that pain evaluation should be applied routinely in clinical assessments of older people with sarcopenia.

The results of the present study show that chronic pain is associated with sarcopenia in Greek community-dwelling older adults. Our findings regarding the relationship between chronic pain and sarcopenia risk are supported by the findings of previous studies that also reported associations between chronic pain, sarcopenia and functional impairment [11,32,33,34]. Regarding chronic pain, in a systematic review conducted by Chen et al. [15], results showed that older adults with chronic pain have a significantly higher prevalence of sarcopenia and risk of developing sarcopenia as compared to those without pain.

Regarding the severity of pain, the results record higher pain severity in sarcopenic participants as compared to nonsarcopenic participants (4.6 ± 1.6 vs. 2.9 ± 1.4; *p* ≤ 0.001). A study conducted on a sample of older English people showed that higher pain intensity was associated with a higher risk of sarcopenia [30].

A possible explanation may be that pain may indicate inflammation, which contributes to muscle loss [35]. In addition, pain may impair mobility and may also promote a sedentary lifestyle [16]. Chen et al. highlighted that pain in the lower back or lower limbs may affect the mobility of older adults and that it is associated with a higher likelihood of developing sarcopenia [15]. These possible mechanisms [e.g., reduced activity and chronic inflammation] require further investigation through large-scale clinical studies in the future. Sarcopenia might also lead to the development of chronic pain through several endocrine, metabolic and immune pathways [36]. However, the question of whether chronic pain increases the risk of sarcopenia or whether sarcopenia exacerbates pain has not been clearly answered and also requires further investigation [15].

In the present study, the majority of the sarcopenic participants reported chronic knee pain. Knee joint pain may cause reductions in muscle strength and affect quality of life. The decline in muscle strength may be because older adults are unable to perform a maximal voluntary contraction due to pain experienced [36]. Knee pain can be caused by various musculoskeletal pathologies, such as osteoarthritis [37,38,39], and may affect gait characteristics [40]. However, in the present study, we did not collect data on the etiology of pain. It would be of great significance to conduct research in order to examine the association of sarcopenia, osteoarthritis and chronic pain.

The level of the association between chronic pain and sarcopenia across different studies may depend on participants’ heterogeneity (e.g., participants hospitalized or not, the average age of the participants, different socioeconomic conditions, etc.) and the different diagnostic criteria for sarcopenia. For example, the results of a systematic review showed that the correlation between chronic pain and sarcopenia was more pronounced in low- and middle-income countries than in high-income countries. One possible explanation is that older adults from low- and middle-income countries often have inadequate healthcare services, which affects the interventions for chronic pain [15]. In the present study, the participants were community-dwelling, older adults. The majority of them were members of the Open Care Centers for the Elderly, indicating that they were active. These centers provide social support and preventive medical services to the elderly who stay at their homes [41]. There are studies that demonstrate no correlation between sarcopenia and chronic pain [42,43] or no correlation between sarcopenia and mild pain [31]. This may be explained by the use of prescribed medication, which can have a crucial role in the link between sarcopenia and pain [15].

The results of the present study also demonstrate a strong association between chronic pain, the number of drugs and comorbidities. These results are reasonable, taking into account the fact that multimorbidity and polypharmacy are highly prevalent in older adults with chronic pain [43]. Pain is prevalent in older adults and is often associated with various medical conditions, making clinical decisions more complex [4,43,44,45]. For example, musculoskeletal disorders (e.g., arthritis) are conditions that significantly impair the health of older adults and are associated with pain [46]. In addition, the literature shows that there is a significantly increased risk of polypharmacy and higher numbers of medications in people with sarcopenia compared with individuals without this condition [47,48]. The association of comorbidities and polypharmacy with chronic pain in patients with sarcopenia needs to be studied further.

An association was also recorded between pain and anxiety and depression. Chronic pain is a common comorbidity of a depressive or anxiety disorder [49]. A possible explanation is that impaired functioning caused by pain can lead to social isolation and increased attention towards threats [49,50]. Depression and anxiety disorders also share the same pathophysiological pathways as pain (e.g., inflammatory processes) [51,52]. Chronic pain and depression may be based on common neuroplasticity mechanism changes, inflammatory processes and psychosocial factors [53].

### 4.1. Clinical Importance of This Study

The diagnostic criteria for sarcopenia do not include an evaluation for pain. Clarifying the relationship between sarcopenia and pain may be of help in the treatment of sarcopenia [54]. The findings of this study highlight the need for prioritizing the assessment and early detection of chronic pain in older people and the need for early pain interventions in the management of sarcopenia in order to help these patients [11].

The results also seem important because the problem of chronic pain imposes a significant economic burden on patients, health services and societies [55]. Chronic pain in older adults may increase healthcare costs and decrease the quality of life of those who experience it [1,56]. Therefore, an effective approach to pain management is, thus, needed for community-dwelling, older adults with chronic pain [1].

### 4.2. Limitations

This study has several limitations. First, information about the causes of pain and its treatment was not collected. Further research is required in this area. In addition, the timeline for the development of sarcopenia and chronic pain could not be established. Further prospective cohort studies examining the association between sarcopenia, pain-related factors and treatment outcomes in older community-dwelling adults with chronic pain are required.

## 5. Conclusions

According to the findings of this study, the prevalence of chronic pain with sarcopenia was high among Greek community-dwelling older adults. Chronic pain was associated with, sarcopenia, comorbidities, the number of drugs and HADS. Thus, healthcare professionals should design prevention and therapeutic programs that take into account both chronic pain and sarcopenia.

## Figures and Tables

**Table 1:** Participants’ characteristics (*n* = 314).

Variable	All Participants (*n* = 314)	Sarcopenia (*n* = 61)	No Sarcopenia (*n* = 253)	*p* Value
		Number, SD		
Age (years)	71.3 ± 7.4	74 ± 6.9	71 ± 7.3	≤0.05
Drugs (number)	2.8 ± 1.5	3.7 ± 1.3	2.4 ± 0.9	≤0.001
Comorbidities (number)	2.9 ± 1.4	3.2 ± 1.0	1.9 ± 1.0	≤0.001
BMI (kg/h^2^)	25.8 ± 3.5	22.8 ± 2.5	26.5 ± 3.3	≤0.001
HGS (kg)	21.1 ± 6.1	15.5 ± 2.5	22.4 ± 6.2	≤0.001
SMMI	7.4 ± 1	7.0 ± 0.7	8.4 ± 0.7	≤0.05
4 m test	4.24 ± 3.0	4.68 ± 0.9	4.1 ± 0.94	0.01
Pain (yes)	66 (21.01%)	27 (44.3%)	42 (16.53%)	≤0.001
Calf measurement	34.9 ± 2.3	32.8 ± 2.1	35.4 ± 2.1	≤0.001
HADS	13.9 ± 2.3	18.4 ± 1.1	12.6 ± 4.0	≤0.001
		Number, percentage		
Gender				
Men	70 (22.3%)	7 (11.5%)	63 (24.9%)	
Women	244 (77.9%)	54 (88.5%)	190 (75.1%)	≤0.001
Falls history				
Yes	82 (26.1%)	31 (50.8%)	51 (20.15%)	≤0.001
Smoking				
Yes	50 (15.92%)	26 (15.3.%)	24 (9.44%)	NS

BMI: body mass index; SMMI: skeletal muscle mass index; HGS: hand grip strength; 4 m test: 4-meter test; NS: non-significant.

**Table 2:** Pain assessment among Greek community-dwelling older adults.

	Nonsarcopenic Participants with Pain (*n* = 253)	Sarcopenic Participants with Pain (*n* = 61)	*p* Value
Pain (Yes)	42 (16.53%)	27 (44.3%)	≤0.001
Pain site			
Knee pain	25 (9.88%)	20 (32.78%)	≤0.001
Lumbar pain	26 (10.27%)	20 (32.78%)	≤0.001
Neck pain	19 (7.5%)	16 (26.22%)	≤0.001
Headache	4 (1.58%)	0 (0.%)	NS
Elbow/wrist pain	2 (0.79%)	1 (1.63%)	NS
Ankle	1 (0.39%)	0 (0.%)	NS
Single-site pain	25 (59.53%)	16 (26.22%)	≤0.001
Multi-site pain	17 (40.47%)	11 (18.03%)	≤0.001
Pain severity (VAS scale)	2.9 ± 1.4	4.6 ± 1.6	≤0.05

NS: non-significant.

**Table 3:** Logistic regression analysis identifying the factors associated with the presence of chronic pain.

Variable	OR	95% CI	*p* Value
Sarcopenia diagnosis	−4.4	−0.89–(−0.00)	≤0.05
Comorbidities	−0.18	−0.34–(−0.02)	≤0.05
Number of drugs	0.12	0.09–0.16	≤0.001
HADS	0.19	0.11–0.31	≤0.05

OR: Odds Ratio; HADS: Hospital Anxiety and Depression Scale.

## Data Availability

The data that support the findings of this study are available from the corresponding author upon reasonable request.
